# Structure, Morphology, and Permeability of Cellulose Films

**DOI:** 10.3390/membranes12030297

**Published:** 2022-03-04

**Authors:** Igor S. Makarov, Lyudmila K. Golova, Galina N. Bondarenko, Tatyana S. Anokhina, Evgenia S. Dmitrieva, Ivan S. Levin, Valentina E. Makhatova, Nazym Zh. Galimova, Gulbarshin K. Shambilova

**Affiliations:** 1 A.V. Topchiev Institute of Petrochemical Synthesis, Russian Academy of Sciences, 29 Leninsky Prosp., 119991 Moscow, Russia; glk@ips.ac.ru (L.K.G.); bond@ips.ac.ru (G.N.B.); tsanokhina@ips.ac.ru (T.S.A.); dmitrievaes@ips.ac.ru (E.S.D.); levin@ips.ac.ru (I.S.L.); 2 Department of Chemistry and Chemical Technology, Kh. Dosmukhamedov Atyrau University, Atyrau 060011, Kazakhstan; mahve@mail.ru (V.E.M.); gnazym@gmail.com (N.Z.G.); shambilova_gulba@mail.ru (G.K.S.)

**Keywords:** cellulose, N-methylmorpholine-N-oxide, biobased membrane, cellophane, permeability, rejection, structure

## Abstract

The work is focused on the study of the influence of the cellulose type and processing parameters on the structure, morphology, and permeability of cellulose films. The free volume of the cellulose films was evaluated by the sorption of n-decane, which is a non-solvent for cellulose. The structural features of the membranes and their morphology were studied using X-ray diffraction, IR spectroscopy, SEM, and AFM methods. The characteristic features of the porous structure and properties of cellulose films regenerated from cellulose solutions in the N-methylmorpholine-N-oxide (NMMO) and cellophane films were compared. Generally, cellulose films obtained from solutions in NMMO have a higher permeability and a lower rejection (as measured using Orange II dye) as compared to cellophane films. It was also found that the cellulose films have a higher ultimate strength and modulus, whereas the cellophane films are characterized by higher elongation at break.

## 1. Introduction

Among plenty of methods of cellulose membrane production, the most interesting are those that do not affect the production of cellulose derivatives (excluding the viscose process), composites based on them, polymer modification, etc. Castings of paper from fibrous cellulose and glassine from finely dispersed cellulose are the best known and available methods of forming cellulose membranes [1]. The development of the chemistry of cellulose solutions has made another method of obtaining cellulose membranes available, such as the formation of films (membranes) from spinning solutions [2]. Historically, the most popular method of producing cellulose films (membranes) today remains the viscose process. Sources of cellulose for the viscose process are wood, flax, hemp, cotton, and others. The main requirements for the used cellulose are the content of an alpha fraction of at least 90% and a degree of polymerization of more than 600 [3,4]. Below, we briefly consider the main stages of the process of obtaining spinning products. The cellulose is dissolved in alkali in a process known as mercerization. It is aged for several days. The mercerized pulp is treated with carbon disulfide to make an orange solution called viscose, or cellulose xanthate. The viscose solution is then extruded through a slit into a bath of dilute sulfuric acid and sodium sulfate to reconvert the viscose into cellulose [5]. The film is then passed through several more baths, one to remove sulfur, one to bleach the film, and one to add glycerin to prevent the film from becoming brittle [6]. The films obtained by this process have received the trade name “Cellophane ™” [7].

In spite of the extensive studies in the field of synthetic membrane polymers and the growing manufacturing rate of these materials, the interest in cellulose films, which have been used in membrane applications for several decades, still persists. The starting point for the application of cellulose films as membranes is probably a work by Freda Wilson [8], which was published as early as 1927 and demonstrated the possibility of sterilization of cellophane films used for dialysis. Since the 1940’s, cellophane films have become widely used in the process of hemodialysis [9]. However, the insufficient rate of blood purification by cellophane membranes led to patients’ discomfort and suffering from pain. This inspired the search for new ways of cellulose dissolution and membrane processing. Scientific search in the following years finally led to invention of cuprophane material, which is obtained via the cuprammonium solution and has higher strength and filtration coefficient than cellophane [10].

The discovery of N-methylmorpholine-N-oxide (NMMO), a novel direct solvent for cellulose [11,12], and the development of the industrial process of fibers (films, membranes, and others) production from solutions in NMMO (MMO or lyocell process), opened an opportunity for the development of a new type of cellulose material. According to BISFA (International Bureau of Standardization of Man-Made Fibres (Brussels, Belgium)) [13], fibers and films prepared via the MMO-process are called Lyocell materials. It is worth noticing that the MMO-process is competitive not only with the environmentally hazardous viscose production process but also with the cuprammonium process (due to complicated reagent regeneration and economic issues of the latter one) [14].

Abe et al. demonstrated [15,16] that cellulose films obtained by the MMO-process can be used as membranes for hemodialysis akin to cellophane or cuprophane films. The limited solubility of cellulose in many solvents makes it possible to use hydrated cellulose films for the separation of water emulsions of aliphatic hydrocarbons, water-alcohol mixtures, etc. [17,18]. Another example of the use of cellulose membranes is the process of removing low molecular weight substances (soda) from aqueous alkaline solutions of hemicelluloses (conventional or Donnan dialysis) [19,20,21].

Cellulose-based nanofiltration membranes are not only used in the medicine and chemical industries, but water purification has also become an important area for the use of such materials. In this case, the cellulose film acts as an integral part of the composite membrane (for example, a false one, providing the mechanical integrity of the membrane) [22]. In order to improve the separating (transport) properties, chemical modification of the surface of cellulose membranes is possible. For example, in [23], a method for modifying cellulose membranes through interfacial polymerization of amino-functional piperazine and 1,3,5-trimesoyl chloride on top of a porous bamboo membrane was proposed. Thus, the unique properties of cellulose provide a high demand for membranes made on its basis in various fields of application.

With reference to the properties of cellulose membranes, one cannot avoid the issue of their structural organization, which is responsible for these features. The structures of the native cellulose powders and cellulose fibers have been described in detail. That is why, from this point onwards, it is expedient to consider the structure of regenerated cellulose. Lyocell fibers have larger crystallites and higher degrees of crystallinity and orientation [24] in comparison with the ones obtained by the viscose process. It was found that the difference in properties is due to the different structure and morphology of these two types of cellulose materials, including their porous structure and pore size distribution. It was recently shown that the pore size of cellulose fibers depends on the drying conditions, as well as on its history [25,26,27].

Thus, in the freshly spun fibers, there were small pores between elementary fibrils. During the drying of fibers, transformation of the pore system was observed. Small pores collapsed in some places, while the pore size increased in other places [25]. The pore size during the transition from a wet to a dry state changed several times, so the pore length increased from 36 nm to 270 nm [26]. The misorientation angle varied from 19° for only spun fibers after solvent removal to 13° for the same samples after drying. If the dried samples were wetted again, the angle increased to 24° [27,28].

Fibers (films) formed in the aqueous precipitation baths are characterized by a monolithic morphology. At the same time, it can be divided into a core and a shell, which are characterized by different orderings [29]. The fiber shell contains larger pores compared to the core [30,31]. Hence, the structure of cellulose membranes can be controlled not only by varying the concentration of the spinning solution [32], the drawing ratio, composition and temperature of the spinning [33,34] and washing bath [28], etc.), but also at the final stage—drying [25,26,27].

The successive drying and wetting of cellulose materials with a liquid in order to change their structure and morphology is reflected in the so-called “activation” of cellulose membranes. McBain and Kistler demonstrated that cellophane films are almost impermeable to alcohols [18,35]. The low permeability of cellophane for a number of organic solvents (methanol, ethanol, acetone, etc.) was obtained in refs. [36,37]. The thickness of the cellophane films had practically no influence on the results. To improve the permeability, the authors suggested a procedure consisting of the swelling of the initially dry cellophane films in water followed by the replacement of water with the liquid to be tested. Such an “activation” of the films provides a significant growth of the film’s permeability up to the values sufficient for membrane applications.

While the formation of fibers by the MMO-process and their properties have been studied quite extensively, there are only very scarce, and often contradictory, data on the structure and morphology of cellulose films. Therefore, the aim of this work is to carry out a comparative study of structure, morphology, sorption, permeability, and mechanical properties of various cellulose films depending on the cellulose nature and way of their preparation.

## 2. Materials and Methods

### 2.1. Materials

Sulfate viscose cellulose produced by Baikal pulp and paper mill (Baykalsk, Russia) with degree of polymerization of 750, equilibrium moisture content of 8% and alpha-cellulose content of 94% (Russian state standard GOST 6840-78) [38].

Sulfate cellulose produced by Kotlas pulp and paper mill (Kotlas, Russia) with degree of polymerization of 650, equilibrium moisture content of 8% and alpha-cellulose content of 90%.

The degree of polymerization of cellulose was determined according to Russian state standard GOST 9105-74 [39].

Commercially available cellophane film (thickness of 30 ± 1 μm) produced by viscose production process in accordance with Russian state standard GOST 7730-89 [40] with degree of polymerization of 200 (C-1), and cellophane film kept in ethanol and warm water (50 °C) and dried to equilibrium moisture content (C-2) (glycerol free).

N-methylmorpholine-N-oxide with melting point of 100–120 °C (corresponds to 8–10 wt% moisture content) supplied by DEMOCHEM (Shanghai, China) was used as a solvent for cellulose. To suppress thermos-oxidative we used 0.5 wt% of propyl gallate (Sigma-Aldrich, St. Louis, MO, USA).

The ethanol used in this work contained 4 wt% of water and was not further desiccated.

To assess the rejection of membranes an anionic dye Orange II (Sigma Aldrich, USA) with molecular weight of 350 Da, maximum absorbency band wavelength of 483 nm, and solubility parameter δ = 29.2 MPa^1/2^ was used.

n-decane (Component-Reactive, Russia) with density of 0.735 g/cm was used to measure the sorption capacity of cellulose films.

### 2.2. Cellulose Dissolution

To prepare the cellulose solutions in NMMO the “solid phase dissolution” method developed earlier [41] was applied. The procedure consists of the following steps:

- Mixing of cellulose, propyl gallate and NMMO powders taken in the required ratio;
- Mechanical activation of the system by intensive shear deformation in the compressed state. This results in formation of H-complexes [42];
- Transformation of the solid phase activated cellulose—NMMO system to melt by heating up to 110–120 °C

Single screw SCAMIA extruder (SCAMIA, Saumur, France) was used for the last two steps. Boetius optical microscope (VEB Kombinat Nadema, Ruhla, former GDR) was applied to check the quality of the prepared solutions.

### 2.3. Films Preparation

All cellulose films were made from 12 wt% cellulose solutions in NMMO by means of HLCL-1000 Coater Laminator (ChemInstruments Inc., Fairfield, OH, USA) operating at 120 °C. The thickness of the prepared films was controlled by setting the gap value between the rolls of the laminator. To avoid sticking of the formed film to the laminator rolls siliconized PET and polyimide films having were used as backing and release liners.

Immediately after preparation of the laminate as described above the release liner was removed from one side and the film of cellulose solution in NMMO on the backing was immersed into the coagulation bath (the temperature of the coagulation bath was 21 ± 1 °C) and kept there for 24 h. Water was used as a coagulating medium in all experiments. In the coagulation bath NMMO is washed out completely from the film while cellulose film is being coagulated.

After solvent removal the cellulose films were dried in the device preventing film shrinkage and enabling uniform film tension at drying. The drying proceeded until equilibrium moisture content (8 ± 1 wt%) is achieved.

The labeling of the samples prepared via MMO-process is shown below as follows (Type of cellulose feedstock—film labelling): Baikal pulp and paper mill (LF-1), Kotlas pulp and paper mill (LF-2), cut cellophane film (LF-3). The initial cellophane film is further referred to as C-1 sample and cellophane without plasticizers (glycerol) (C-2).

### 2.4. Films Characterization

The X-ray diffraction study of the prepared dry cellulose films was performed by means of Rigaku Rotaflex RU-200 diffractometer (Rigaku Corporation, Tokyo, Japan) equipped with a rotating copper anode, linear focus 0.5 × 10 mm, source operating mode 50 kV–100 mA, characteristic CuKα radiation wavelength λ = 1.542 Å, secondary graphite was used monochromator, horizontal D-Max/B goniometer and scintillation detector). X-ray scanning was recorded at room temperature in reflection mode according to the Bragg-Brentano scheme in the continuous θ–2θ scan mode in the angular range of 5–45°, at a speed of 2°/min and at a scanning step of 0.04°.

IR-spectra of dry and wet films were registered by HYPERION-2000 (Bruker Optics, Ettlingen, Germany) IR-microscope equipped with ZnSe crystal, in the range of 600–4000 cm^−1^. The resolution was 2 cm^−1^ (150 scans).

The morphology of the dried cellulose films was studied on a Philips SEM 505 scanning electron microscope (Philips Industries, Eindhoven, The Netherlands) equipped with a tungsten cathode, updated modern capture system, and computer image processing MicroCapture SEM 3.0 M and on a FEI Scios microscope (FEI Company, Hillsboro, OR, USA) at an accelerating voltage of less than 1 kV in the secondary electron mode [43]. Cleavages were made after freezing in liquid nitrogen. Images of the sample’s surface were obtained using an atomic force microscope Solver P47 AFM (NT-MDT, Moscow, Russia).

To evaluate the porosity of the cellulose films n-decane was used, which is a liquid able to penetrate into the cellulose films while not dissolving cellulose [44]. The porosity was calculated from the amount of n-decane absorbed by a film.

Nanofiltration characteristics of the cellulose membranes were studied in the stainless-steel dead-end filtration cells equipped with magnet stirrers at trans-membrane pressure of 20 atm as described earlier [45]. The effective membrane area was 33.2 cm^2^. The volume of the testing liquid was selected so that no more than 20% of it came across the membrane during the experiment. The pressure in the filtration cell was provided by helium.

The permeate flux was measured by weight using “Sartorius” electronic balances (Sartorius AG, Goettingen, Germany) with resolution of 0.001 g. The receiver for the permeate was designed so as to minimize volatile solvent evaporation. The permeability of the membranes (*P*) was calculated as follows:

(1) $$P=\frac{m}{S\cdot \Delta t\cdot \Delta p}$$

Here m (kg) is the weight of the permeate that came across the membrane with surface area S (m^2^) during the period of time equal to Δ*t* (hours) at trans-membrane pressure of Δ*p* (bar).

Rejection (**R**) was used to characterize the separation efficacy of the membranes, as follows:

(2) $$R=(1-\frac{c_{P}}{c_{0}})\cdot 100\%,$$

where *C*_0_ и *C_P_* are concentrations of a dissolve substance in the initial testing solution and in the permeate, respectively.

The mechanical properties of the cellulose films were studies using TT-1100 (ChemInstruments, Fairfield, OH, USA) and Instron 1122 (Instron, Norwood, MA, USA). The films for tensile testing were cut into stripes. The initial distance between grips.

## 3. Results

**Figure 1:**
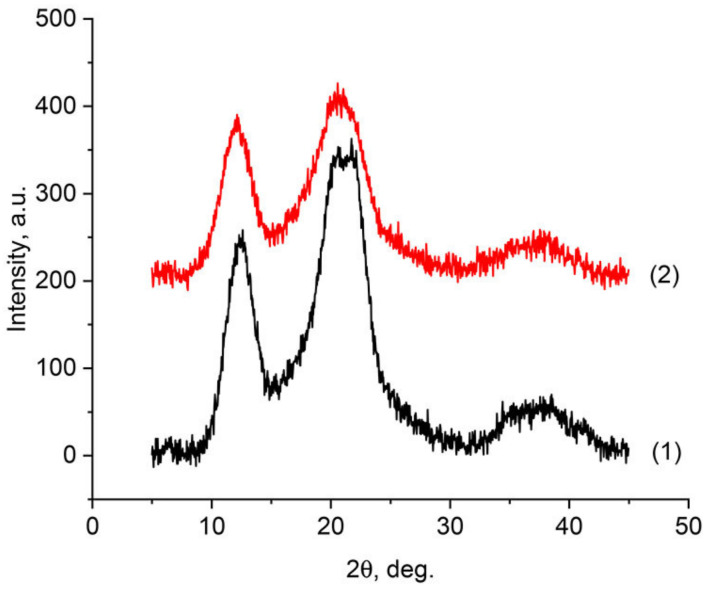
Diffractograms of the initial cellulose film C-1 (1) and lyocell-type film LF-1 (2).

Regardless of the method of membrane formation, the process of obtaining spinning solutions and their regeneration is accompanied by cardinal structural changes. These transformations are associated with a change in the conformation of cellulose chains. X-ray diffraction analysis makes it possible to evaluate these polymorphic transformations. The X-ray diffractograms of the initial cellophane films C-1 and lyocell-type film LF-1 are presented in Figure 1, curves 1 and 2 respectively.

A distinctive feature of native cellulose is the presence of three characteristic peaks in the diffraction patterns in the region (~14.6°, ~16.6°, and ~22.7°), which correspond to polymorph I [46]. As can be seen from Figure 1, the observed diffraction patterns for samples LF-1 and C-1 are similar in appearance. At the same time, the diffraction pattern of regenerated cellulose is fundamentally different from that observed for polymorph I. The main reflections in the diffraction patterns are in the reflection region 2θ~12.1°, 2θ~20.1°, and 2θ~21.5°, and refer to the planes (101), (101), and (002), respectively [47]. The observed structure of regenerated cellulose refers to the cellulose II polymorph [48]. An assessment of the crystallinity of the obtained samples made it possible to reveal that in the case of samples LF-1, it reaches 60%, and for industrial cellophane, it does not exceed 56%. The obtained values of crystallinity are in good agreement with the data already presented in the literature for viscose and Lyocell fibers. And they allow us to talk about a more perfect structural order in the LF-1 films compared to the C-1 samples.

**Figure 2:**
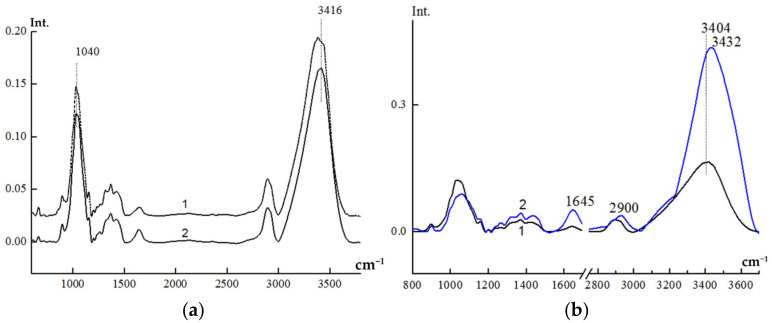
Comparison of IR-spectra for dry films C-1 (**a**) 1 and LF-1 (**a**) 2 and dry LF-1 film (**b**) 1 and after swelling in water (**b**) 2.

The IR-spectra of the same films are shown in Figure 2.

Qualitatively, the IR-spectra of these films are almost identical. In the spectrum of C-1, one can see some splitting of the most intensive valents—OH and C-O group’s vibrations (3416 and 1040 cm^−1^, respectively). This indicates the less ordered structure in the cellulose structure of the cellophane film.

To access the degree of ordering in the cellulose materials the 2900 cm^−1^(νCH) and 1370 cm^−1^ bands are often used as internal standards (the latter band relates to complex vibrations including the deformation of –OH, –CH, and –CH_2_ groups). The ratio of intensities of the 1370/2900 bands is the value most sensitive to the degree of crystallinity of cellulose. Table 1 shows the crystallinity indexes calculated from this ratio and O’Connor’s crystallinity indexes calculated on the basis of the 1430/900 band intensities ratio. The 1430 cm^−1^ band relates mainly to the deformational vibrations of CH_2_ groups, whereas the 900 cm^−1^ band is connected to amorphous fragments of the cellulose structure, so this value is thought to be most sensitive to the side group’s ordering [49].

The data presented in Table 1 demonstrate that the crystallinity indexes are higher for the lyocell film LF-1 than for the cellophane one (rows 1–3 of the Tab). The indexes of conformational ordering (rows 5 and 6) are also higher for the LF-1 film, whereas the index of the amorphous phase content (row 7) is higher for the cellophane film.

**Figure 3:**
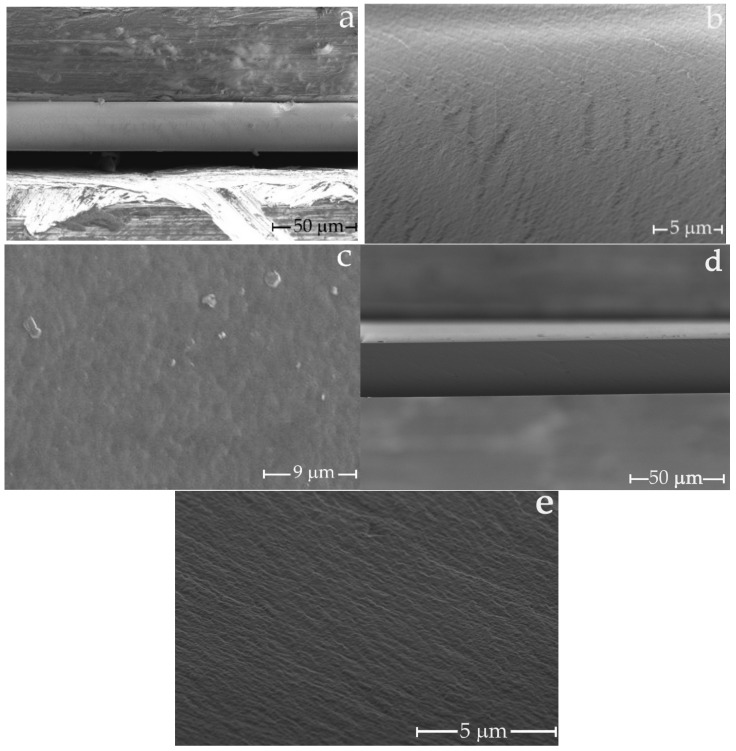
SEM microphotographs of LF-1 and C-1 films: (**a**,**b**) are cross-sections of LF-1, (**c**) is surface layer of LF-1, (**d**,**e**) are cross-sections of C-1.

The morphology of the cellulose films was studied by means of SEM and AFM. Figure 3 represents the SEM images of the surface and cross-section of the LF-1 and C-1 films.

In [17,50], the authors describe the surface of the cellulose film observed by electron microscopy at 8000 and 5000 magnifications as smooth, without visible pores. On the other hand, [16,51] fixes a rough surface. The average pore diameter in the first paper is 7.7 nm, and in the second it varies in the range of 50–179 nm. As could be seen from photos in Figure 3, both its surface and cross-section possess prominent roughness, which could be correlated with its structure and porosity. The microstructure of the transverse cleavage of the cellulose membrane is symmetric, dense (homogeneous), and practically defect-free. The formation of such a morphology for cellulose membranes is of particular interest. Since the cellulose membranes (LF-1) were successfully prepared using the non-solvent induced phase separation methodology, where a rigid precipitant water was used as a coagulant, it is important to note that the air-dried LF-1 membrane has smooth edges. Probable defects caused by sample drying do not appear on the surface. Unfortunately, SEM microphotographs did not provide information on the pore size in the C-1 and LF-1 membranes.

**Figure 4:**
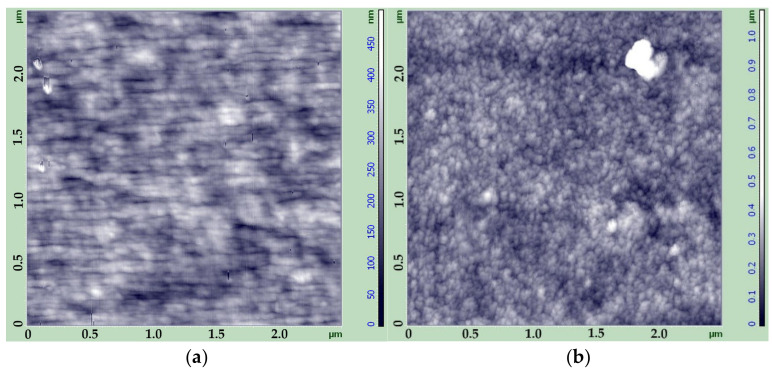
AFM images of the surface of films C-1 (**a**) and LF-1 (**b**).

AFM provided a better insight into the fine film’s structure. Figure 4 demonstrates the structure of the surface of the C-1 and LF-1 films.

As can be seen from the images, the cellophane film is characterized by the profound orientation of the small structural elements with a height below 450 nm. On the contrary, LF-1 film has a uniform cellular structure with no evident orientation, and the height of the surface structure elements (most probably microfibrils) is up to 1000 nm. This leads to an uneven, rough morphology of the surface. It can be supposed that the different morphology of these two types of cellulose films leads eventually to a difference in their porous structure and permeability.

The free pore volume of the cellulose film can be estimated by the sorption of inert liquids. In our work, we used n-decane, which is capable of filling in the pores in the cellulose but unable to cause swelling due to its hydrophobic nature. Along with cellophane (C-1, C-2) and cellulose (LF-3) films were investigated sorption properties of the cellulose films LF-1 and LF-2, obtained from the wood cellulose produced by various methods. The obtained data are shown in Table 2.

Polymer membranes are subject to high pressure gradients, so mechanical properties are of the utmost importance for their successful application. The results of the tensile tests for the films under investigation (tensile strength, Young modulus, and elongation at break) are summarized in Table 2.

As can be seen from the presented data, cellulose films LF-1 and LF-2 have the highest tensile strength and modulus, while cellophane film C-1 is more elastic (elongation at break is about 65%). We believe that the higher tenacity of LF-1 samples compared to C-1 is caused by the higher degree of crystallinity of the LF-1 membrane, as confirmed by X-ray diffraction and IR spectroscopy. Conditioning of the cellophane film in ethanol leads to leaching of the plasticizer and other additives. This results in a 53% decrease in the modulus and an even more dramatic drop in elongation at break. At the same time, the tensile strength grows from 40, up to 60 MPa.

The minimum sorption was observed for the cellophane film C-1. Surprisingly, the LF-1 film demonstrated higher sorption in spite of its higher crystallinity and trend to fibrillation. The LF-3 film, which was obtained from the cellophane film dissolved in NMMO, is characterized by even higher sorption, most likely due to the lower cellulose degree of polymerization in the initial cellophane film. Higher sorption of LF-2 film obtained from sulfate cellulose produced by Kotlas pulp and paper mill with α-cellulose content of 90% can be attributed to a lower degree of ordering in the initial cellulose. But the highest n-decane sorption was observed in the case of C-2 cellophane film, which was treated with ethanol and water. In all probability, treatment by ethanol results in washing out various additives that are present in the commercially available cellophane, which leads to increased sorption capacity of the sample.

The described experiments confirm the difference in the structure and morphology of cellophane films and those obtained via the MMO-process. It was, however, found out that, in spite of this difference, both of these types of films have very low permeability for water–ethanol mixtures.

Taking into account the application of cellulose membranes in nanofiltration, it was interesting to explore the evolution of the structure of different types of cellulose films when swelling in water.

Figure 2 represents the IR-spectra of the initial (dry) LF-1 film and of the same film after swelling in water for 30 min at room temperature. Table 1 demonstrates the indexes of ordering of LF-1 and C-1 films after swelling in water, calculated on the basis of relative intensities of characteristic bands (data for dry films in Table 1 also). The content of the bound water in the film can be assessed by the relative intensity of the 1645 cm^−1^ band (the last column in Tab).

Analyzing the data on spectral indexes of ordering (Table 1), one may draw the following conclusions: Increasing water content at swelling promotes alignment of the side groups, which leads to a dramatic increase in O’Connor’s crystallinity index (column 1). Its value grows monotonously with the water content. The overall crystallinity also increases (columns 2 and 3), whereas the overall amorphous phase content decreases (column 7). The trend of the conformational ordering is not so obvious. It can be supposed that water penetrating predominantly into amorphous regions promotes straightening of the cellulose chains and alignment of the side groups, thus increasing crystallinity and decreasing the amorphous phase content. This may lead to appearance of new conformations at the border between crystalline and amorphous phases which results in the ambiguity in the conformational indexes. The increase in crystallinity was greater at the swelling of the cellophane film than in LF-1, although the water content in it is fairly low. The possible explanation consists of the fact that the content of amorphous and pseudo-crystalline phases in cellophane is higher, so all incoming water goes into these regions, causing ordering of the structure through alignment of the side groups.

It can be summarized that preliminary swelling leads to structural changes that may result in the growth of the cellulose film’s permeability. As was demonstrated in [37,52], the permeability of the swollen films can be several times higher than that of untreated ones. Therefore, the cellulose films were kept in water before being placed into the nanofiltration cell.

The permeability of ethanol and the nanofiltration of the Orange II dye were measured for the cellulose films under investigation. All tests were performed until steady values of the parameters were obtained. The resulting steady-state values of the film’s permeability and rejection are summarized in Table 2. As can be seen from this Table, the best filtration characteristics were obtained for the cellophane films. The ethanol permeability for C-1 is 0.11 kg/(m^2^∙h∙atm), which is almost twice as much as ethanol permeability for commercially available DuraMem 150 crosslinked polyimide membrane. The value of the rejection (67%) is comparable to that of DuraMem 150 [53]. After conditioning in ethanol and water (C-2 film), the ethanol permeability increases considerably, whereas the rejection deteriorates. This could be connected to the changes in the pores’ size and structural rearrangements described above.

Unlike cellophane, the films prepared from NMMO solutions (LF-1 and LF-2) have much lower rejection than Orange II and higher ethanol permeability. This fact highlights once more the principal difference in structure between the cellophane and films obtained by the MMO-process. It is worth noticing that LF-3 film obtained through dissolving cellophane in NMMO has the highest permeability and lower rejection than LF-1, probably due to the low degree of polymerization of the initial cellophane and the structuring of cellulose in the NMMO solution.

**Figure 5:**
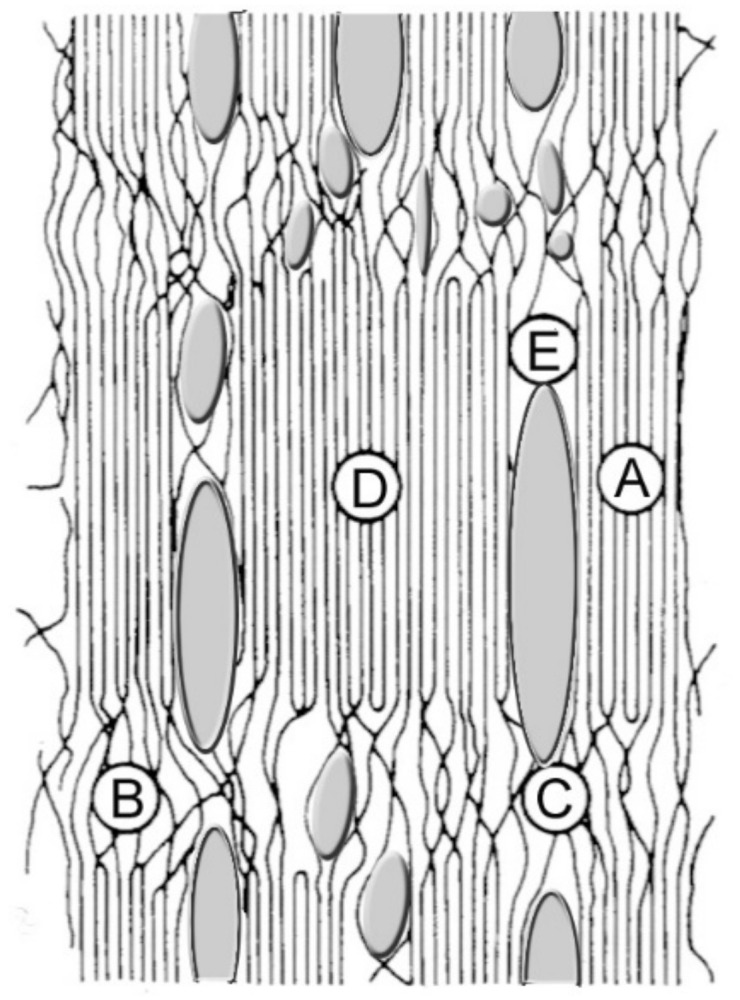
The scheme of morphology and porous structure of cellulose films prepared by MMO-process: **A**—crystallites, **B**—amorphous regions, **C**—strands, **D**—clusters, and **E**—pores.

The obvious difference in the sorption capacity of the samples should be related, in our opinion, to the peculiarities of their structure. Cellophane films obtained by the viscose process have a lower degree of crystallinity and smaller crystallites as compared to the cellulose films prepared by the MMO-process. Their structure is therefore quite uniform, with a rather narrow pore size distribution. On the contrary, the structure of cellulose regenerated from solutions in NMMO is characterized by a higher degree of crystallinity and larger crystallites. Furthermore, the crystallites are organized into anisometric clusters and fibrils with corresponding anisometric spaces (pores) between them. The scheme of the pore’s distribution in this type of film is presented in Figure 5.

Water removal at drying leads to rearrangement of H-bonds, which results in the collapse of the pore system and low permeability of films. The main difference between cellophane and films prepared by the MMO-process consists of the fact that cellophane contains a lot of small pores, whereas NMMO films comprise a smaller number of larger pores. In accordance with these reasons, dry lyocell films have a lower porosity than cellophane. At swelling, the porous structure is partially restored, which leads to an increase in sorption capacity and permeability.

## 4. Conclusions

Therefore, the complex study of the structure, morphology, permeability, and mechanical properties of cellulose membranes prepared by viscose and NMMO properties from cellulose of various origins demonstrated the following:

- The structure and morphology of films depend greatly on the method of their preparation [54] and the type of the cellulose used. The structure of lyocell films is completely different from that of cellophane;
- The application of a “rigid” water precipitator makes it possible to form a uniform, dense morphology in membranes formed by the MMO process.
- Water removal at drying leads to rearrangement of H-bonds, which results in the collapse of the pore system and low permeability of films;
- The effect of water activation on the structure of membranes obtained through the viscose and MMO processes was revealed. It is shown that for cellophane, the values of O’Connor’s crystallinity index are higher than for membranes formed from solutions in NMMO;
- Cellulose films obtained from solutions in NMMO are characterized by higher ethanol permeability and lower Orange II rejection as compared to cellophane films;
- This can be connected to the non-uniform porous structure of the lyocell films, with plenty of large pores;
- Lyocell membranes have higher tensile strength and modulus, whereas cellophane films have higher deformability and elongation at break.

The results should be taken into account when preparing and using the cellulose membranes in various organic media. Further studies on the preparation of new cellulose membranes from solutions in NMMO suggest varying the conditions for their formation. For example, changing the chemical composition of precipitation and washing baths, drying methods, etc., will make it possible to control the membrane structure and, as a result, the transport properties of membranes.

## Figures and Tables

**Table 1:** Relative intensities of characteristic bands in the IR-spectra of dry LF-1 and C-1 cellulose films and indexes of ordering of swollen C-1 and LF-1 films based on relative intensities of characteristic bands in the IR spectra.

№	Relative Intensities	Films			
		Dry		Swollen	
		LF-1	C-1	LF-1	C-1
1	D_1430_/D_900_ (O’Connor’s crystallinity index)	1.64	1.14	2.54	3.45
2	D_1370_/D_2900_	0.87	0.85	0.90	0.88
3	D_1430_/D_2900_	0.68	0.65	0.71	0.75
4	D_1321_/D_2900_	0.69	0.68	0.80	0.81
5	D_1265_/D_2900_	0.32	0.30	0.51	0.54
6	D_1152_/D_2900_	0.71	0.66	0.70	0.63 ^1^
7	D_900_/D_2900_	0.52	0.58	0.28	0.22
8	D_1640_/D_2900_	0.38	0.21	0.91	0.82

^1^ Band appears as a shoulder, the exact value of the intensity is difficult to determine.

**Table 2:** Ethanol permeability and Orange II rejection for water activated membranes, mechanical characteristics and sorption data of n-decane by cellulose for dry membranes.

Films	n-Decane Sorption, wt% *	Rejection, %	Permeability, kg/(m^2^∙h∙bar)	Tensile Strength, MPa	Young Modulus, GPa	Elongation at Break, %
C-1	2	67	0.11	40	1.7	65
C-2	30	29	0.23	60	0.9	9
LF-1	5	5	0.5	74	2.5	10
LF-2	12	8	1.18	53	1.4	5
LF-3	8	6.5	7	15	0.3	28

* The sorption of n-decane was calculate as the ratio of the film increase in weight to the initial weight of the film.

## Data Availability

The data presented in this study are available on request from the corresponding author.
